# A comprehensive review on the impact of calcium and vitamin D insufficiency and allied metabolic disorders in females

**DOI:** 10.1002/fsn3.3519

**Published:** 2023-07-26

**Authors:** Aftab Ahmed, Muhammad Awais Saleem, Farhan Saeed, Muhammad Afzaal, Ali Imran, Sidra Akram, Muzzamal Hussain, Aqsa Khan, Entessar Al Jbawi

**Affiliations:** ^1^ Department of Nutritional Sciences Government College University Faisalabad Faisalabad Pakistan; ^2^ Department of Human Nutrition and Dietetics Mirpur University of Science and Technology Mirpur Pakistan; ^3^ Department of Food Sciences Government College University Faisalabad Faisalabad Pakistan; ^4^ Agricultural Extension Directorate, MAAR Damascus Syria

**Keywords:** calcium, metabolic disorders, polycystic ovary syndrome, vitamin D deficiency

## Abstract

Calcium is imperative in maintaining a quality life, particularly during later ages. Its deficiency results in a wide range of metabolic disorders such as dental changes, cataracts, alterations in brain function, and osteoporosis. These deficiencies are more pronounced in females due to increased calcium turnover throughout their life cycle, especially during pregnancy and lactation. Vitamin D perform a central role in the metabolism of calcium. Recent scientific interventions have linked calcium with an array of metabolic disorders in females including hypertension, obesity, premenstrual dysphoric disorder, polycystic ovary syndrome (PCOS), multiple sclerosis, and breast cancer. This review encompasses these female metabolic disorders with special reference to calcium and vitamin D deficiency. This review article aims to present and elaborate on available data regarding the worldwide occurrence of insufficient calcium consumption in females and allied health risks, to provide a basis for formulating strategies and population‐level scientific studies to adequately boost calcium intake and position where required.

## Graphical abstract

This review encompasses these female metabolic disorders with special reference to calcium and vitamin D deficiency. This review article aims to present and elaborate on available data regarding worldwide occurrence of insufficient calcium consumption in females and allied health risks, with the aim to provide a basis for formulating strategies and population‐level scientific studies to adequately improve calcium intake and status where required.

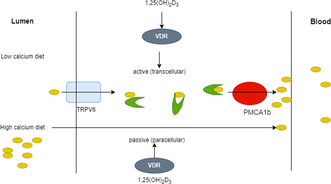

## 1 INTRODUCTION

Vitamin D's significance for musculoskeletal maintenance including rickets management has indeed been traced back to at least the 1930s. Within the influence of vitamin D, calcium is effectively absorbed through the small intestines. To mineralize as well as bone strength, calcium and phosphorus combine to generate hydroxyapatite crystals. As a result, healthy bone mineralization necessitates a diet high in the amalgamation of vitamin D and calcium. Vitamin D's primary job is to improve calcium‐absorbing performance in the intestine; however, over the last few decades, various atypical applications for vitamin D have been researched. Vitamin D receptors (VDR) could be identified in a diversity of tissues outside the skeleton and small intestine, including type 1 T helper cells, neutrophils, glands, the brain, as well as various organs (Grant & Boucher, 2019).

Vitamin D insufficiency threatens about half of the global community (Greene‐Finestone et al., 2017). Vitamin D insufficiency disturbs an approximated 1 billion individuals worldwide, crossing all races and age groups (Cashman, 2020). This outburst of hypovitaminosis D is fundamentally owing to behavioral and ecological variables that bound sunshine contact, which is compulsory for UVB‐induced vitamin D production in the skin. Because black people's melanin absorbs more UVB than white people's, they necessitate longer sunlight time to manufacture the equivalent magnitude of vitamin D (Rusińska et al., 2018).

Since hypovitaminosis D is an independent prognostic predictor intended for whole death in women, the significant incidence of vitamin D scarcity is the foremost community healthcare alarm (Chakhtoura et al., 2018). Vitamin D might help prevent breast cancer, cardiovascular disease, osteoporosis, autoimmune diseases, allergy, diabetes, premenstrual syndrome, PCOS, preeclampsia, and hypertension in women, according to new findings (Pittas et al., 2019; Manson et al., 2019; Jiang et al., 2020).

The drop in estrogens following menopause causes greater bone turnover, a diminution in bone mineral density, and an accelerated likelihood of fracture. The incidence of metabolic and cardiovascular illness rises, mental disorders are common, and musculoskeletal problems may reduce the quality of life. Additionally, alterations in physical structure, such as a rise in abdominal fat and a reduction in lean muscle, elevate the likelihood of vitamin D insufficiency. On the other side, vitamin D shortage might make menopausal‐related illnesses and pain worse (Pilz et al., 2012; Salehpour et al., 2012).

Cross‐sectional findings indicate that menopause is linked to increased weight and reallocated fat mass. Following menopause, women experience a loss of fat‐free mass, get a tendency to work out less, and experience a significant rise in abdominal fat (Lovejoy et al., 2008). Such incidence of obesity, particularly abdominal fat deposition, raises the likelihood of cancer, metabolic and heart disease, and overall mortality (Wehr et al., 2011). Numerous investigations exposed that overweight and low vitamin D concentrations are related. But it has long been unclear whether obesity is a result of vitamin D inadequacy or perhaps it is the etiology of it. This problem is clarified by a new bidirectional Mendelian randomized investigation. This study suggests that a lower 25(OH)D results in a higher BMI; however, any effects of a lower 25(OH)D rising BMI are expected to be limited (Vimaleswaran et al., 2013).

Premenopausal women do not often experience cardiovascular events, and the gender disparity between cardiovascular occurrences in young men and women might result from endogenous estrogen's supposed preventive role. The decline in concentrations of estrogen after menopause may be the cause of the detrimental alterations in lipid and glucose metabolism that occur at this time and contribute to an elevation in the frequency of cardiac incidents (Collins et al., 2007; Strange et al., 2015). Numerous prospective types of research revealed indications connecting lesser vitamin D values to both cardiac risk indicators and cardiac incidents (Pludowski et al., 2013).

While the actual cause of musculoskeletal problems remains unknown, estrogen deficiency is sometimes blamed for them. Although estrogen does not directly affect the articular components that might produce knee pain, it does have a significant antinociceptive impact on inflammation and the brain processing of nociceptive information (Felson & Cummings, 2005). Because of the naturally declining estrogen concentrations throughout menopause, women are more expected to develop osteoporosis and fractures (Gurka et al., 2016).

Calcium is a mineral to facilitate an imperative responsibility in a variety of bodily processes (Cormick & Belizán, 2019; Bourassa et al., 2022). Even though the impacts of dietary calcium otherwise calcium supplements have indeed been directed toward approaching various medical goals recently, studies on the significance of calcium have mostly concentrated on bone development. The association between calcium ingestion and preeclampsia throughout pregnancy was first noticed in the 1980s (Belizan & Villar, 1980). This came from a study of the Mayan diet in Guatemala, which consisted of soaking and heating corn utilizing limewater before processing, and elevated calcium consumption that resulted was linked to a lower incidence of preeclampsia and eclampsia (Gomes et al., 2022).

Osteoporosis is another of the most common medical concerns among women nowadays, with more than 41 million women expected to be afflicted within the coming 20 years if existing tendencies continue. Physicians should concentrate on osteoporosis management instead of only treating the disease. The preponderance of bone mass is built throughout adolescence and earlier adulthood, with about 90% of skeletal mass deposited mostly by the age of 18. According to recent studies, young women's calcium consumption is much lower than the recommended dietary allowance (Wallace, 2019).

It is impossible to address calcium without mentioning Vitamin D. Vitamin D is requisitely designed for calcium absorption inside the body. Obtaining the vitamins and minerals that are needed is usually done through foodstuffs. However, merely a handful of foods, such as mushrooms, asparagus, milk, milk products, calcium, and vitamin D‐fortified items, possess vitamin D and calcium. Vitamin D is mainly obtained by exposing your skin to sunshine (15–20 min each day) or by taking supplementations. Supplementation with vitamin D3 might be recommended for persons who are vitamin D insufficient and cannot receive it through sunlight or diet. The physiology and metabolism of vitamin D and calcium would be the theme of this appraisal. Briefly highlight mechanisms of thought to underpin vitamin D–calcium correlation and their clinical consequences, with a special emphasis on women's health.

Many studies have identified dietary supplements that may help women to overcome metabolic disorders and support normal postmenopausal recovery. The purpose of this review is to inform readers of the most recent scientific studies on the effects of vitamin D and calcium on disorders including obesity, hypertension, premenstrual dysphoric disorder, polycystic ovary syndrome (PCOS), multiple sclerosis, breast cancer, and other possible health‐related benefits of these nutrients. So, when necessary, women can boost their consumption of calcium and vitamin D.

## 2 METABOLISM OF VITAMIN D AND CALCIUM

### 2.1 Metabolism of vitamin D

Vitamin D is alienated into two categories: vitamin D2 named ergocalciferol and vitamin D3 also named cholecalciferol. Irradiated fungus or yeast is used to make ergocalciferol. Cholecalciferol is generated in the skin and could be present in fatty fish like salmon and mackerel. Vitamin D could be administered to fortify foods in both types, although only cholecalciferol can be produced endogenously in the skin. When 7‐dehydrocholesterol, a molecule found in the skin, is irradiated to UVB photons within the wavelengths of 290 and 315 nm, it is transformed into vitamin D3 which isomerizes to generate vitamin D3. Skin color, age, sunscreen application, the period of the day, seasonality, and altitude can all influence the quantity of vitamin D3 produced in the skin (Blau & Collins, 2015). Sources of foods abundant in vitamin D are shown in Table 1.

**TABLE 1:** Sources of foods abundant in vitamin D.

Food	Portion	Vitamin D international units (IUs)
Fish liver oil	1 tbsp	1350
Swordfish (cooked)	3 oz	565
Salmon (cooked)	3 oz	445
Tuna (cans with liquid/evaporated)	3 oz	155
Vitamin D‐fortified orange juice	1 c	136
Milk (vit D fortified)	1 c	120
Yogurt (fortified by 20% of recommended dietary intake of vit D)	6 oz	80
Sardines (cans with oil/dry)	2 oz	45
Beef liver (cooked)	3 oz	40
Yolk of egg	1	40
Cereal (fortified by 10% of recommended dietary intake of vit D)	1 c	60

Abbreviations: c, cup; oz, ounce; tbsp, tablespoon.

**FIGURE 1:**
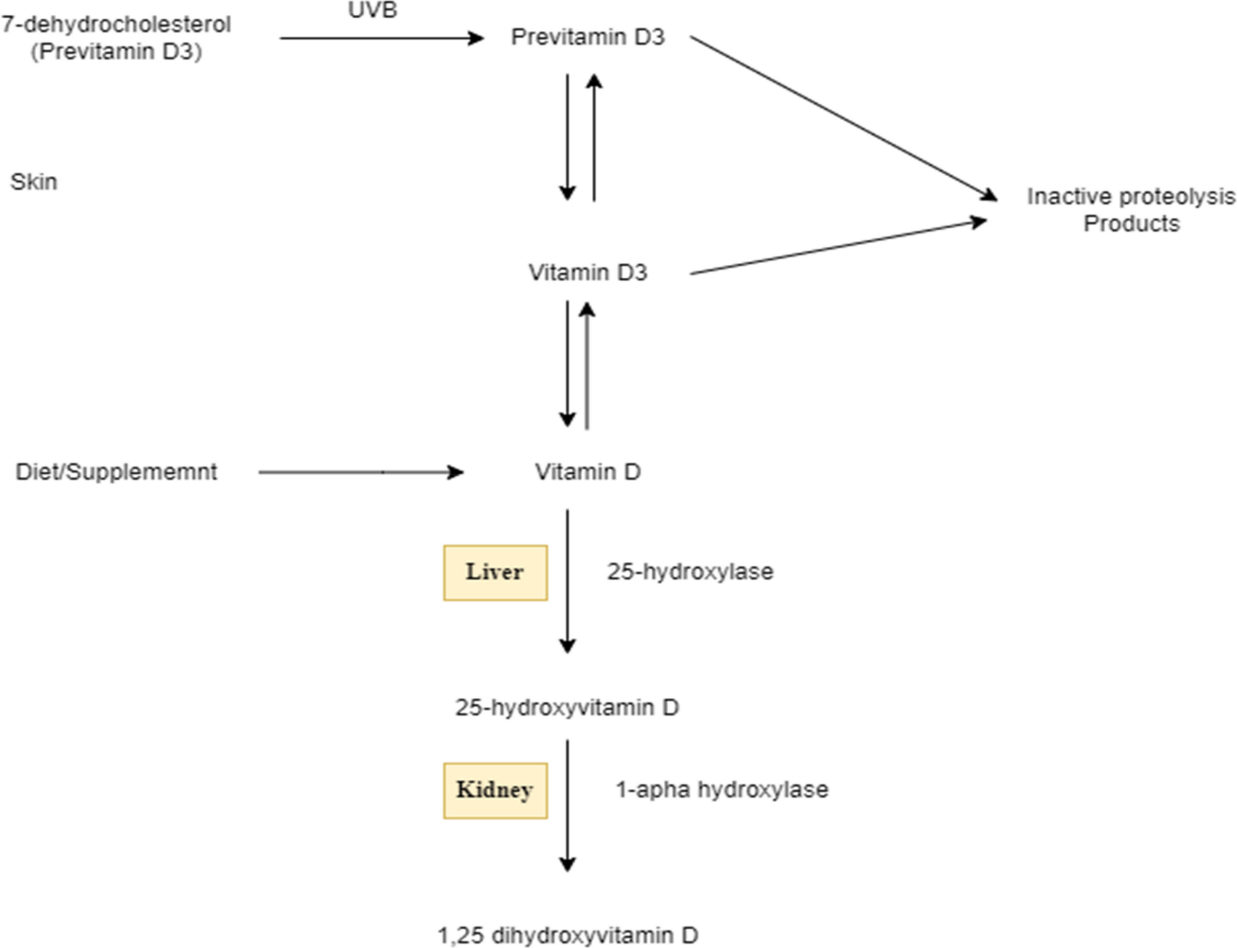
Vitamin D metabolism; intermediary versions of the vitamin require ultraviolet radiation and metabolism in liver and kidney, latter of which is partially regulated by PTH.

Vitamin D enters the bloodstream attached to vitamin‐D‐binding protein whenever it is produced in the skin (D3) or received from food (D2 or D3). This combination is delivered to the liver, wherein vitamin D is hydroxylated in the 25 places to generate 25‐hydroxyvitamin D (25[OH]D), which is subsequently hydroxylated in the one position by 1‐α‐hydroxylase to generate the hormonal form of vitamin D, 1,25‐dihydroxy vitamin D (1,25[OH]2D) in the kidney as shown in Figure 1, 25(OH) 2D joins the desired cell obligated to vitamin‐D‐binding protein, adheres to the vitamin D receptor (VDR) in the cytosol, after which joins the nucleus and heterodimerizes with the retinoic acid X binding site to boost transcription of vitamin‐D‐dependent genetic makeup essential for bone metabolism, calcium uptake, as well as other nonclassical features (e.g., suppression of genes essential for tumor development) (Tangpricha et al., 2005).

The 24‐hydroxylase catabolizes circulatory 1,25(OH) 2D to generate 1, 24, 25(OH) 2D, a deactivated vitamin D molecule. By boosting the transcription of the 24‐hydroxylase, 1,25(OH)2D promotes its catabolism. Anticonvulsants, for example, have recently been discovered to promote 1,25(OH)2D catabolism by stimulating the pregnane X receptor, culminating in additional production of the 24‐hydroxylase (Zhou et al., 2006).

### 2.2 Calcium absorption in the intestine

Vitamin D's main job is to improve calcium and phosphorus amalgamation in the intestine so that bone mineral composite can develop properly. A paracellular or transcellular pathway transports calcium throughout the intestine. The paracellular pathway is primarily a passive activity, while 1,25(OH) 2D plays a significant role within the transcellular process. Several calcium transport proteins that are VDR govern intestinal calcium absorption. TRPV6 (transient receptor potential cation channel, subfamily V, member 6) is a calcium stream found on the enterocyte's luminal side. This channel allows calcium to penetrate enterocytes more easily. When contrasted to jejunum and ileum, TRPV6 is extremely controlled by 1,25(OH) 2D and is particularly abundantly activated inside the duodenum.

**FIGURE 2:**
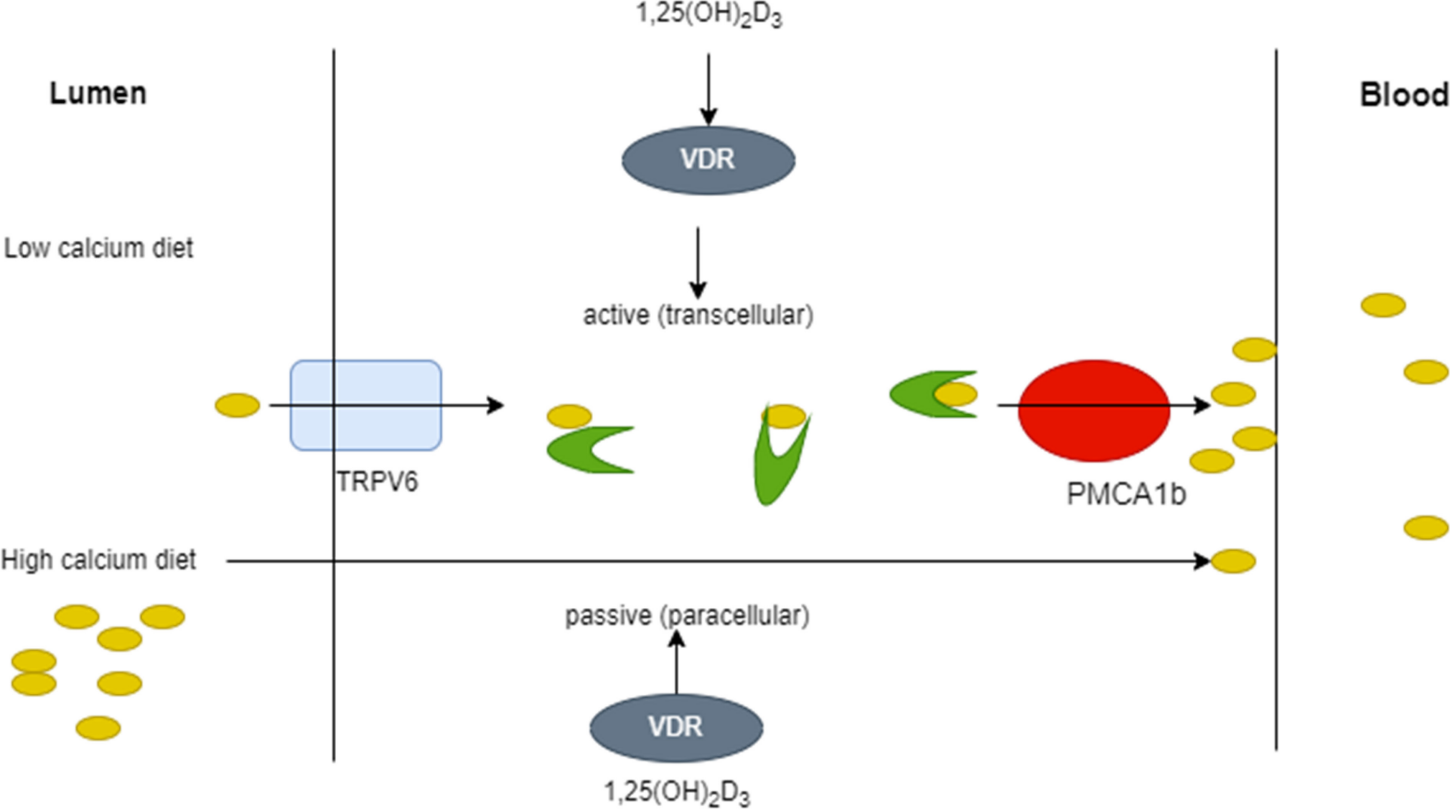
A potential calcium absorption technique.

TRPV6 levels decrease with aging in women, therefore, helps to understand why calcium absorption decreases with age (Pattanaungkul et al., 2000). Calbindin 9 k is a calcium transport protein complex that transports calcium from the enterocyte's luminal side to its basal membrane. Ultimately, on the basal membrane of the enterocyte, two more vitamin D‐regulated cation interchange proteins, PMCA1b (plasma membrane calcium ATPase, which is mainly expressed in the gut, is most likely the calcium‐regulated pump that pushes the calcium out from the intestinal epithelium into the bloodstream), and NCX1 (the sodium–calcium exchanger is a sarcolemmal protein that is essential for maintaining calcium homeostasis in the heart), occur to export calcium into the bloodstream as shown in Figure 2 (Walters et al., 2007).

To improve the effectiveness of calcium absorption, sufficient vitamin D amounts are required. Lacking enough vitamin D, the body only absorbs about 12% of the calcium it consumes. In the presence of adequate vitamin D, gastrointestinal calcium absorption rises from 30% to 40% (Holick, 2007). Vitamin D receptor‐deficient mice produce osteomalacia in a rat model that could be restored with a recovery diet high in calcium and lactose. This research confirms that vitamin D's primary function is to improve intestinal calcium absorption, which is compulsory for strong bone mineralization (Christakos et al., 2011). Sources of foods abundant in calcium are shown in Table 2.

**TABLE 2:** Sources of foods abundant in calcium.

Food	Portion	Calcium (mg)
Cereal with fortification	½ c	250–650
Cheddar cheese	1 oz	200
Jack Cheese	1 oz	215
Eggnog	1 c	325
Fish	3 oz	320
Buttermilk	1 c	290
Dry Milk	¼ c	200
Fat‐free milk	1 c	300
Whole Milk	1 c	280
Soy or rice milk with fortification	1 c	350
Tofu with calcium fortification	¼ c	210
Yogurt	8 oz	350
Dried beans	½ c	80
Figs	5 (medium size)	130
Greens, collards	½ c	140
Orange juice, calcium fortified	½ c	190
Soybeans	½ c	130
Spinach	½ c	130

Abbreviations: c, cup; oz, ounce.

## 3 REVIEW METHODOLOGY

In the current manuscript, we conducted a view of peer‐reviewed from 1995 to 2022 that linked calcium and vitamin D with women's health. Furthermore, the relevant literature was examined one by one in detail. The literature search was carried out using scientific databases comprising Science Direct, Scopus, Google Scholar, PubMed, Library Genesis, Science Hub, and Cochrane Library using the following subject headings; Calcium, vitamin D deficiency, metabolic disorders, oxidative stress, inflammation, obesity, type 2 diabetes mellitus, hypertension, and PCOS. Finally, the authors collected the latest available literature from primary and secondary sources.

## 4 EFFECTS OF CALCIUM AND VITAMIN D ON THE REPRODUCTIVE STAGE OF WOMEN

### 4.1 Function of vitamin D

Exploration of the possessions of vitamin D on ovarian reserve indicators, namely, the anti‐Mullerian hormone (AMH), is scarce (Jukic et al., 2015). Throughout the development of follicles, granulosa cells in females create the gonadal‐specific glycoprotein known as AMH. AMH is a cooperative prognostic marker in assisted reproductive technology (ART) since it is generated by expanding ovarian follicles and does not alter substantially throughout the menstrual cycle. Even though investigations have exposed that environmental variables like vitamin D insufficiency could change AMH expression and blood concentrations, AMH is still one of the strongest screening indicators for ovarian reserve (Merhi et al., 2014). In research to determine if 1,25(OH)2D3 influences AMH mRNA expression, vitamin D therapy led to a dose‐related reduction in AMH mRNA levels in granulosa cells. Follicle‐stimulating hormone (FSH) mRNA expression rates and cell proliferation, on the other side, were found to be higher (Wojtusik & Johnson, 2012).

AMH concentrations could be 17% fewer in the winter than in the summer. The concentration of vitamin D is also somewhat lower around this time of year. According to published research, females' AMH and 25(OH)D level variations correlate (Dennis et al., 2012). A favorable correlation between blood vitamin D values and AMH values was discovered in a cross‐sectional investigation of 379 premenopausal females with normal menstrual cycles. In adult females (>40 years), vitamin D inadequacy is accompanied by a reduced ovarian reserve (Merhi et al., 2014). When AMH values are employed in clinical assessment, vitamin D insufficiency must be considered (Dennis et al., 2012).

Cholesterol is used to make all sex hormones. The action of several enzymes regulates the synthesis of sex hormones. According to recent research, vitamin D may have an impact on how these enzymes are expressed and function (Lundqvist, 2014). Another investigation focused on how vitamin D affected the granulosa cells' ability to produce progesterone. It found that vitamin D increased progesterone synthesis and 3‐b‐hydroxysteroid dehydrogenase mRNA expression, which in turn stimulated ovarian steroidogenesis (Merhi et al., 2014). Estrogen is a precursor to androgens, and estrogen production is catalyzed by the enzyme aromatase (estrogen synthetase). It is articulated in a diversity of tissues, counting adipose tissue, the hepatic, the brain, and the mammary. Vitamin D may alter the expression of aromatase in particular tissues (Usluogullari et al., 2015).

### 4.2 Calcium's role in early female reproductive events

More than seven million oogonia have transformed into primary oocytes throughout fetal maturation. The oocytes are kept in meiotic arrest (prophase I) from birth until adolescence when a tiny population is stimulated to develop once every cycle. When luteinizing hormone (LH) levels are high, the granulosa cells that surround the oocyte produce growth factors that aid in oocyte maturation. Oocytes that lack calcium will not fully complete meiosis I, even though the precise role of calcium in oocyte maturation has been debated. Additionally, calcium injection is a prerequisite as well as a substantial requirement for meiosis to resume in vitro (Jamnongjit & Hammes, 2005). It was discovered that L‐type calcium channels contribute to the calcium influx before nuclear maturation (Tosti, 2006). Furthermore, calcium oscillations inside the oocyte are necessary for the development of crucial characteristics that give the oocyte the capacity to become triggered in retort to sperm (Machaca, 2007). It is broadly acknowledged that calcium oscillations perform a crucial part in the activation of mouse oocytes during fertilization and subsequent development. The development of the blastocyst was not influenced by the suppression or stimulation of calcium signaling, and instead implantation, fetal growth, and longevity, accordingly. These results show how calcium signaling has significant effects on implantation and even postimplantation growth (Ozil et al., 2006). Although each egg needs the right intracellular calcium signaling for fertilization to occur, it must be highlighted that species‐specific differences do occur. While most mammals produce numerous wavelike calcium oscillations after fertilization, other species, including frogs, fishes, sea urchins, and many others, only produce one calcium transient (Ellinger, 2016).

## 5 VITAMIN D, CALCIUM, AND PREGNANCY

Calcium supplements throughout pregnancy have also been looked into. Kids whose mothers participated in the calcium group exhibited a lower incidence of elevated hypertension at 7 years of age than kids whose mothers participated in the placebo group, according to the follow‐up of kids whose mothers were in the calcium group (Cormick et al., 2019). According to a literature review, kids whose moms took calcium supplements had a lower SBP of 1.90 mm Hg from the age of 1–9 years (Jiang et al., 2020).

Progeny of rats for whom the mothers experienced low calcium consumption throughout pregnancy had SBP levels of 11.9 mmHg greater than the progeny of rodents for whom mothers had a typical calcium eating plan, according to a study that followed them until they were 52 weeks old (Villa‐Etchegoyen et al., 2019). Kids for whom mothers participated in the calcium group had a 25% lower probability of acquiring tooth decay at 12 years of age than kids for whom the mothers belonged in the placebo group, according to the RCT (Bergel et al., 2010).

### 5.1 Vitamin D scarcity in pregnancy: Its importance and prevalence

The enlistment of maternal calcium treasury is obligatory for fetal musculoskeletal growth throughout pregnancy. These results in several physiologic adjustments, counting improved motherly bone enlistment and intestinal calcium administration that are at least moderately regulated by 1,25‐dihydroxy vitamin D. In the first trimester, overall, 1,25‐dihydroxy vitamin D levels rise, while free 1,25‐dihydroxy vitamin D levels rise in the last trimester, before reversing to baseline all through the peripartum phase and lactation (Richard et al., 2017).

Renal synthesis, which is exalted by prolactin and placental lactogen, seems to serve the main essential role, albeit 1‐hydroxylase is also expressed in the placenta, decidua, and fetal kidneys (Lopes et al., 2016). Its value notes that vitamin D is almost entirely absorbed as a type linked to plasma protein and that vitamin‐D‐binding protein concentrations ascend all through pregnancy. Despite this, concentrations of free 1,25‐dihydroxy vitamin D are raised all through pregnancy. In women with vitamin D inadequacy, maintaining adequate mineral homeostasis through pregnancy devoid of jeopardizing maternal or fetal bone mass could be complicated (Gallo et al., 2020; McAree et al., 2013).

### 5.2 Fetal complications

As previously stated, the fetus is reliant on the mother's 25‐hydroxyvitamin D and calcium reserves; thus, elevated incidence of vitamin D insufficiency in pregnancy has consequences for the progeny of concerned mothers (Principi et al., 2013; Walsh et al., 2013). Vitamin D levels in the neonate's bloodstream are roughly 60%–70% of maternal levels (Brooke et al., 1980). In Canadian research, 45% of newborns delivered to females who gained vitamin D substitutes throughout pregnancy had vitamin D inadequacy (classified as cord blood concentrations of vitamin D 10 ng/mL), with differences linked to seasonality and color of skin (Richard et al., 2017).

Several longitudinal types of research have linked the prevalence of short‐for‐gestational age (SGA) delivery to vitamin D deficiency (Burris et al., 2012). Vitamin D levels under 10 ng/mL in the middle trimester be linked to a thrice increased likelihood of SGA in a large prospective analysis, despite no substantial, ongoing link between vitamin D and birth weight (Burris et al., 2012). Vitamin D insufficiency at 13 weeks gestational age showed linked to reducing newborn weight and an elevated probability of SGA in one more multi‐ethnic prospective cohort of above 2900 births (Leffelaar et al., 2010).

Low birth weight has been allied to an amplified risk of heart disease and type 2 diabetes in adulthood (Kaijser et al., 2008). Despite the probable link between hypovitaminosis D throughout pregnancy and SGA babies, the protracted effects are still to be thoroughly explored. Just 30% of the kids of women who were tested for hypovitaminosis D in later pregnancy persisted for follow‐up at age 9 years in the largest UK cohort yet investigated on the subject (Gale et al., 2008). These children's cardiovascular measures were not linked to their mothers' vitamin D levels all through pregnancy (Gale et al., 2008).

Vitamin D insufficiency through pregnancy can impact long‐term growth and bone mineralization in children (Sempos et al., 2021). Vitamin D seems to be connected with femoral development throughout the prenatal phase (Ioannou et al., 2012); at delivery, babies delivered to women having lower vitamin D levels had shorter long bones (Tihtonen et al., 2022). Furthermore, this is not a uniform conclusion, and other investigations contradict the findings (Gale et al., 2008). Although it has been claimed that kids of females with low vitamin D values had decreased bone density (Woon et al., 2019), a current perspective and well‐designed study found no such link (Lawlor et al., 2013). As an outcome, the true impact of mother vitamin D on baby bone health is currently being investigated.

There has also been a considerable study on the impact of maternal vitamin D on children's respiratory diseases. Reduced fetal blood vitamin D levels in newborns showed linked to higher respiratory tract infections by the respiratory syncytial virus (Bradley et al., 2020). Infants whose mothers reported greater vitamin D consumption throughout pregnancy (AlFaris et al., 2019) and when fetal blood vitamin D levels are increased tend to have a decreased occurrence of recurrent wheeze (Camargo Jr et al., 2011). The link to asthma, on the other hand, remains still unknown (Camargo Jr et al., 2011). As a result, newborn respiratory infections seemed to be linked to maternal hypovitaminosis D, although additional research is needed to confirm this (Bradley et al., 2020).

Further atopic symptoms, such as eczema (Krieger et al., 2018) and food allergies (Weisse et al., 2013), might be linked to motherly vitamin D scarcity. In research of 230 newborns tested throughout their first year of life, those with a cord blood vitamin D level < 20 ng/mL had a greater risk of eczema (Jones et al., 2012). As a result, proof suggesting a connection between vitamin D and immunological response is conflicting and understudied.

The data suggesting a link between vitamin D insufficiency and the likelihood of developing type 1 diabetes in kids is also debatable. In the children of mothers with gestational vitamin D levels in the lower quartile, new nested case–control research discovered a double occurrence of type 1 diabetes acquisition (Sørensen et al., 2012). Finnish research, on the other hand, revealed no variations in vitamin D levels among mothers whose kids got type 1 diabetes and mothers whose kids did not (Miettinen et al., 2012).

### 5.3 Maternal complications

Preeclampsia is among the major common maternal problems related to vitamin D insufficiency throughout pregnancy. Numerous longitudinal pieces of research had found that women having insufficient vitamin D levels had a higher chance of preeclampsia (Omotayo et al., 2018). Yet, additional investigations had unable to substantiate this link, especially in high populations (Fogacci et al., 2020). A meta‐analysis of observational investigations conducted in 2013 indicated a link between hypovitaminosis D and the risk of preeclampsia, although the link was not substantial when variables were taken into account. As a result, while the link between hypovitaminosis D and preeclampsia appears plausible, further well‐designed prospective trials, particularly in high‐risk women, are needed to verify it (Aghajafari et al., 2013).

The significance of vitamin D in the establishment of type 2 diabetes and gestational diabetes mellitus (GDM) has also been investigated, and vitamin D insufficiency seems to be associated with changes in glucose homeostasis throughout pregnancy (Aghajafari et al., 2013; Senti et al., 2012). Hypovitaminosis D in early pregnancy or the second trimester is linked to a higher risk of GDM, which appears to be irrespective of the mother's age, ethnicity, or body mass (Parlea et al., 2012). Higher vitamin D sufficiency was associated with a lower likelihood of maternal hyperglycemia at gestational ages 24 and 28 weeks in a cohort of pregnant women whose vitamin D levels have been assessed before 16 weeks of pregnancy, but only among smokers. As a result, vitamin D might be a controllable susceptibility factor for GDM (Tomedi et al., 2013).

Vitamin D insufficiency has also been considered linked to an elevated risk of bacterial vaginosis during pregnancy (Hensel et al., 2011). Bacterial vaginosis is important because it has been shown linked to negative obstetrical consequences such as the preterm burst of membranes, preterm delivery, early labor, postpartum endometritis, and preclinical multiple miscarriages (Martin, 2012). It might additionally have gynecological effects on women's well‐being, such as endometritis, a higher incidence of sexually acquired illness (Martin, 2012), like HIV (Mirmonsef et al., 2012), cervical intraepithelial neoplasia (Gillet et al., 2012), and tubal infertility (Van Oostrum et al., 2013).

Some researchers have found a higher incidence of cesarean section amongst women with hypovitaminosis D (Scholl et al., 2012); however, other research implies that this result is unrelated to maternal vitamin D sufficiency (Fernández‐Alonso et al., 2012). This link must be established since cesarean birth can result in immediate and long‐term consequences for women, including rehospitalization, postpartum infections (Kanatani et al., 2019), deep venous thrombosis, and improper placental insufficiency in subsequent pregnancies (Marshall et al., 2011).

Maternal vitamin D insufficiency in earlier pregnancy was linked to higher levels of depressive symptoms in the Amsterdam cohort, as measured by a questionnaire completed at 15 weeks of pregnancy (Brandenbarg et al., 2012). However, because vitamin D readings were taken so close to the questionnaire delivery, a causal association could not be demonstrated (Brandenbarg et al., 2012). Serum vitamin D levels in early pregnancy were shown to be inversely associated with anxiety ratings in a group of African American mothers (Cassidy‐Bushrow et al., 2012). As a result, preliminary evidence supports a link between anxiety and hypovitaminosis D, but more research is needed (Kiely et al., 2020).

## 6 THE IMPACT OF CALCIUM AND VITAMIN D INSUFFICIENCY ON THE GENERAL HEALTH OF WOMEN

### 6.1 Immune‐modulating properties of vitamin D and calcium

#### 6.1.1 Vitamin D's immunomodulatory property

An intriguing purpose of vitamin D in immunity has been brought to attention by the expression of the VDR in immune cells. Nowadays, a strong collection of clinical data suggests that vitamin D regulates innate and adaptive immunity on a fundamental level. Through intracellular VDR, which is reported to exist in macrophages, T and B cells, natural killer cells (NK), and dendritic cells (DCs), vitamin D has a local immunological action. Vitamin D and the retinoid X receptor (RXR) create a heterodimer following vitamin D binds to its nuclear receptor superfamily member VDR. This compound binds activators and enzymes with histone acetylation affinity and activates the vitamin D response element (VDRE). As a result, the intended gene is regulated as an outcome of the structural alterations in chromatin caused by this combination (Chun et al., 2014).

##### Vitamin D and innate immunity

**FIGURE 3:**
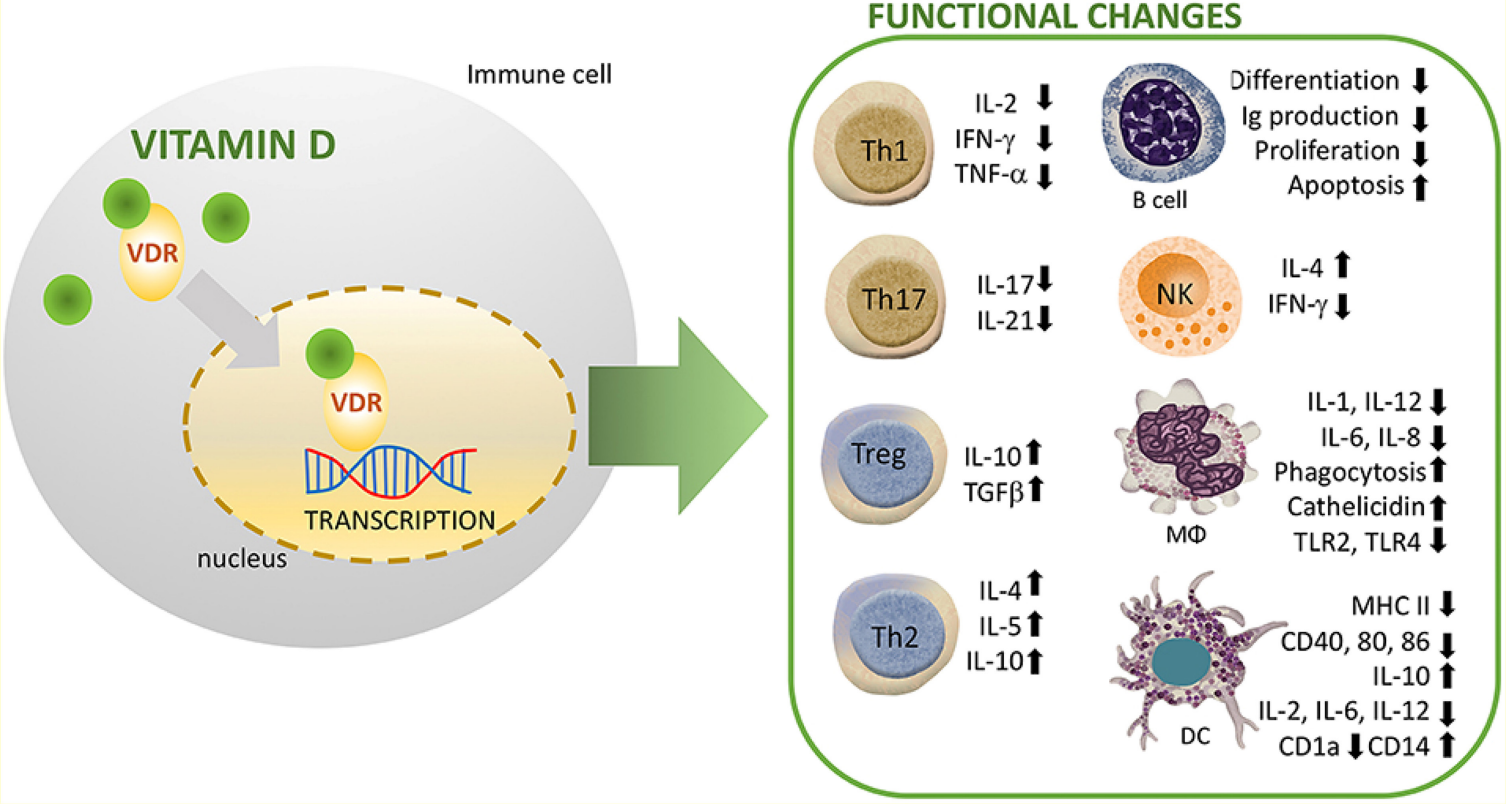
How vitamin D (1,25(OH)2D3) modulates the immune system across a range of immune cell lineages. R stands for receptor; VDR is for vitamin D receptor; M stands for macrophage; NK stands for natural killer (Source; Cyprian et al., 2019).

Vitamin D signaling regulates the production of antimicrobial peptides (AMPs), particularly cathelicidin and defensins, which are variably controlled by innate immunity. The human cathelicidin and defensin 2 gene promoters in this strain include VDRE. The innate immune system's NKT cells are typically derived cells that secrete large volumes of cytokines, such as IL‐4 and IFN‐γ. The appropriate growth and operation of NKT cells are controlled by vitamin D through its interaction with VDR. In this line, IL‐4 and IFN‐γ production was reduced in NKT cells purified from VDR knockout mice. Additionally, vitamin D stimulated NK cell activation (Wang et al., 2004). Chen et al. have investigated how vitamin D administration affects innate immune cells. Likewise, neutrophils and macrophages were shown to produce more IL‐1beta and IL‐8, although their ability to phagocytose was reduced (Chen et al., 2017) (Figure 3).

Parallel findings from other research suggest that vitamin D inhibits the stimulation of macrophages, culminating in the development of an anti‐inflammatory M2 macrophage phenotype (Liang et al., 2019). Interestingly, stimulation of human monocytes with CD40 ligand and IFN‐gamma (IFN‐γ) increased autophagy and antimicrobial peptide production by inducing the transcription of VDR and CYP27B1‐hydroxylase (Klug‐Micu et al., 2013), while vitamin D improves macrophage phagocytosis and bactericidal action against bacteria including P. aeruginosa and M. tuberculosis (Nouari et al., 2016). Moreover, dendritic cells (DCs), which are believed to activate lymphocytes via antigen transmission, had been extensively studied for their immune‐modulating properties of vitamin D as well as its analogs. Current studies revealed that the maturation, differentiation, and longevity of DCs were significantly inhibited in a vitamin‐D‐dependent manner. Several in vitro and in vivo investigations recently showed that defective maturation of DCs is caused by a lower expression level of costimulatory molecules (CD80, CD40, and CD86), MHC class II, and other maturation‐induced surface indicators (Figure 3). Vitamin D significantly hinders the migration and maturation of DCs in exposure to inflammatory events, which leads to decreased antigen‐presenting capability and T‐cell stimulation. Additionally, cytokine flips with enhanced IL‐10 expression and decreased interleukin‐2 (IL‐2) synthesis suppresses the T helper 1 (Th1) phenotype (Figure 3). Consequently, vitamin D and its analogs support the establishment of a tolerogenic condition by keeping DCs in an immature phenotype (Bartels et al., 2010).

##### Vitamin D and adaptive immunity

The VDR is substantially expressed postactivation in both B and T cells, according to early investigations. The actions of vitamin D on T cells should be highlighted as one of vitamin D's primary roles in adaptive immunity. Vitamin D influences CD4^+^ lymphocyte development and stimulation via attaching to the VDR on T cells (Palmer et al., 2011).

A subgroup of CD4^+^ cells called Tregs regulates immunological tolerance and the immune response. Numerous investigations have demonstrated that vitamin D encourages Foxp3^+^ Treg proliferation and effector activities (Mocanu et al., 2013). Vitamin D controls inhibitory T lymphocytes in difficult pregnancies in humans (Ji et al., 2019). Additionally, Treg counts are increased by vitamin D signaling in both inflammatory disorder sufferers and normal individuals (Fisher et al., 2019). It is fascinating to note that vitamin D reduces the transcription of the IL‐2 gene and prevents the generation of pro‐inflammatory Th‐cytokines such as IFN‐γ, IL‐2, and IL‐17 (Figure 3). Immunophenotyping of naive and memory T cells in kids has shown a link between vitamin D and the incidence of infectious diseases, which is consistent with other findings. Elevated vitamin D concentrations were linked to survival in this strain because they resulted in an elevation in memory T cells (Looman et al., 2017). Parallel to this, subsequent research discovered that lower vitamin D values were linked to different T‐lymphocyte stimulation in newborns. The vitamin‐D‐depleted subgroup showed reduced numbers of naive CD4^+^ T cells, CD4^+^ T‐helper cells, and CD8^+^ cytotoxic T lymphocytes, as measured in newborns and mothers' cord blood. Additionally, one in six infants diagnosed with sepsis also had vitamin D deficiency, indicating an increased likelihood of infections in this population (Youssef et al., 2019). Furthermore, T‐cell activation RhoGTPase‐activating protein (TAGAP) and IL‐2RA have been discovered as vitamin‐D‐sensitive genes of CD4 T cells in individuals with MS using a single‐nucleotide polymorphism (SNP) study (Berge et al., 2016).

Likewise, it believes that vitamin D inhibits B‐cell growth and immunoglobulin synthesis. Secondly, it prevents B cells from differentiating into plasma cells. Relatively little VDR is expressed by naive B cells. On the other hand, B‐cell VDR activity rises after stimulation. In the context of pertinent stimuli, vitamin D signaling increases the death of activated B cells. Similarly, vitamin D prevents the development of memory B cells and the release of IgG and IgM immunoglobulins by stimulated B cells (Rolf et al., 2014).

#### 6.1.2 Calcium and the stimulation of immune cells

T‐cell activity depends on calcium homeostasis. Through SOCE, the T‐cell receptor might cause the discharge of intracellular calcium. Purinergic receptors enhance the T‐cell response, whereas TRP channels regulate T cells' performance and VDCC channels control it (Trebak & Kinet, 2019). In this review, Trebak and Kinet (2019) discuss the intricate interaction of calcium channels on and in T cells. Mice are protected against EAE when STIM1 and STIM2 are reduced (Ma et al., 2010). A similar thing was seen in T cells following CaV3.1 channel deletion, together with the modified cytokine release and decreased gene transcription (Wang et al., 2016). Additionally, vitamin D's impact on MS sufferers is presently being studied since they typically have low vitamin D concentrations. In cell culture, vitamin D administration appears to lessen neurotoxicity caused by oxidative stress (AlJohri et al., 2019). Its effects on immune cells have also been studied (Medrano et al., 2018). A reduced amount of vitamin D appeared to be marginally advantageous in EAE rats, whereas vitamin D supplements and consequent hypercalcemia were detrimental (Häusler et al., 2019; Lichtman et al., 1983; Martin et al., 2016).

#### 6.1.3 Vitamin D, calcium, and immune disorders

The making antibodies protect the body from outside invaders while preserving self‐tolerance. In the setting of vitamin D shortage, it tends to be a high sensitivity to viruses and a diathesis, in a biologically sensitive environment to autoantibodies. Vitamin D's basic responsibilities are to sustain calcium homeostasis as well as to enhance bone mass. Vitamin D increases calcium amalgamation inside the intestinal tract and supports osteoclast formation and intracellular reabsorption in bone. Vitamin D also allows the collagen network in the bones to mineralize. Vitamin D is taken from the meal or is synthesized within the dermis. Vitamin D production is determined by geography, seasonal, blocker usage, and hyperpigmentation since it is conditions that promote following ultraviolet irradiation. 1. By engaging with the vitamin D receptor, the activated hormone executes its effects on different tissues (Aranow, 2011).

Multiple sclerosis, rheumatoid arthritis (RA), diabetic mellitus (DM), irritable bowel syndrome, and systemic lupus erythematosus (SLE) are all associated with vitamin D and calcium insufficiency (Adorini, 2005). Decreased plasma volume of vitamin D and calcium has been connected to the expansion of chronic illness in MS and autoimmune diabetes mellitus (DM) (Munger et al., 2006). Insufficient vitamin D consumption in utero has also been allied to β‐cell antibodies (Martens et al., 2020). Decreased in‐utero exposure, as determined by decreased maternal vitamin D absorption during maternity, is related to a dramatically higher risk of pancreatic autoimmunity in mothers whose unborn kid was at risk of having autoimmune DM. Vitamin D has also been implemented to enhance autoimmune disorders proceed.

Much research on vitamin D levels in lupus patients was studied all over the nation (Kamen & Aranow, 2008). Vitamin D levels in individuals are frequently lower than in sick or healthy individuals. Vitamin D insufficiency is still frequent, with even more than half of the lupus individuals receiving low amounts, and acute inadequacy (vitamin D levels G10 ng/mL) is also widespread. Several, but not all, research has revealed that disease outcomes in patients are negatively related to vitamin D status. Squat vitamin D amounts are connected with disease activity and intensity in other autoimmune conditions such as MS and RA (Correale et al., 2009).

### 6.2 Hypertension

In advanced countries, hypertension is the fourth reason for death before its time, and in developing countries, it is the seventh cause. Up until now, the biggest causes of illness and death in Africa were communicable diseases, birth complications, and poor nutrition. In recent years, the focus has shifted to noncontagious diseases, and advanced countries now face what is called a “double burden of diseases.” In the earliest half of the 20th era, hypertension was nearly nonexistent in African cultures. However, evaluations demonstrate that more than 40% of adults in some parts of Africa now have hypertension (Amrein et al., 2020).

After extensive research, the function of calcium in the detection and care of high blood pressure has still been challenged. Over the past few years, the functional significance of calcium in monitoring and controlling hypertension is currently unexplained even after multiple research mediation. When compared to conventional food that is high in fat and saline and poorer in calcium, magnesium, potassium, and fiber, an appropriate diet profile rich in calcium from low‐fat dairy foods, fruit, and vegetables has now been reported to reduce hypertension considerably. Furthermore, timeline and observational studies of epidemiological study information on calcium as a sole vitamin had normally characterized that the efficacy on reducing hypertension is modest, on the order of 2 mm Hg, and the impact on diastolic BP, if there is any, could be much lesser (Hamet, 1995). However, consistent hypertension drop on this scale via calcium intake may even have remarkable cardiovascular preventive care consequences on the local scale. But there have been minimal correlations with both 25(OH) Vit D and hypertension, the brief various project suggested that vitamin D, either as light irradiation contact or as an oral dose, may reduce hypertension (Nam et al., 2017).

Deviations in the modulation of exogenous calcium as altered by the parathyroid hormone, vitamin D, as well as the renin–angiotensin pathways may be associated with calcium metabolite disorders in hypertensive. The consequence of calcium management can be seen under specific physiologic circumstances, like gestation hypertensive, which is particularly categorized by hypocalciuric and massive rises in intracellular stores of calcium. A theory of calcium consumption changes hypertension is confirmed by controlled experiments on dense populations. Regardless of the community assessed losing weight has always been the primary, the most consistent nonpharmacological way of controlling hypertension (Hamet, 1995).

Additional study is required on the combinations of variables that affect hypertension adversely like sodium, body composition, as well as alcohol, and others that might be favorable such as calcium, potassium, and magnesium. The implications of food insecurity trends on long‐term regulating blood pressure, the origins of hypertension, and the curative perspective for hypertension monitoring should all be researched extensively. Irregularities in calcium absorption were found to also have a part in hypertension pathology (Afridi et al., 2008). Individuals with hypertension and minimal vitamin D levels seem to be at a greater risk of cardiovascular problems and strokes. Whereas the overwhelming of vitamin D intervening analyses have utilized adverse health outcomes in their present study, at least a single meta‐analysis of interventions research indicates that vitamin D supplements may lower fatalities (Witham et al., 2009).

### 6.3 Obesity

Obesity is a new health concern that is becoming more and more important. Even though there are many causes of obesity, most of the major causes of the advanced obesity crisis are related to diet. Emerging research shows that calcium and vitamin D may regulate weight growth, especially with an energy‐restricted diet (Song & Sergeev, 2012). Interestingly, several studies have suggested the possible function that dietary calcium, vitamin D, and milk products might have in control of overweight, glucose metabolism, and glucose intolerance. In individuals of any age, authors of prospective studies have observed that weight and body fat are adversely associated with calcium intake (Alloubani et al., 2019).

#### 6.3.1 Vitamin D and body mass

Researchers first thought that there might be an association between vitamin D and being overweight (Al Hayek et al., 2018). They found a link between having more body fat and having low serum 25(OH)D levels. They thought that this was because the fat‐soluble vitamin D was being stored in the fatty tissue. Zemel et al., 2000 stated that getting more calcium in your diet can make you less fat and suggested that calcium affects the production of 1,25(OH)2D. In some research, a strong inverse correlation has been found between a squat level of vitamin D, higher fat mass, and higher jeopardy of gaining weight over time. But it is important to note that obese people have trouble moving around and do not like to do things outside (Lagunova et al., 2011). In these people, their skin may not get enough UVB irradiation to keep their vitamin D levels at a healthy level. It is also significant to say that vitamin D3 is stored inside the adipose tissue of obese people. These must be taken into account when trying to figure out why past studies show a negative link between vitamin D levels and body mass. Numerous randomized controlled studies have studied the outcome of vitamin D supplementation (typically with high dietary calcium consumption) on human fat cells. Caan et al., 2007 demonstrated that 1000 mg Ca^+^ and 400 IU vitamin D per day has a minor result on weight increase, especially in women with low calcium intakes. Taking 600 mg Ca plus 1100 IU of vitamin D increased good changes in HDL cholesterol, TAG cholesterol, and total cholesterol. However, it did not affect body weight (Zareef et al., 2018). RDAs (recommended dietary allowances) of vitamin D and calcium for various age groups of females are shown in Table 3.

**TABLE 3:** RDAs (recommended dietary allowances) of vitamin D and calcium for various age groups of females.

Age (female)	RDA of calcium (mg)	Upper limit (mg)	RDA of vitamin D (IU)	Upper limit (IU)
0–6 m	200	1000	400	1000
6–12 m	260	1500	400	1500
1–3 y	700	2500	600	2500
4–8 y	1000	2500	600	3000
9–13 y	1300	3000	—	4000
14–18 y	1300	3000	—	4000
19–30 y	1000	2500	—	—
31–50 y	1000	2500	—	—
51–70 y	1200	2000	—	—
70+ y	1200	2000	800	—
18 y or younger (pregnant/lactating)	1000	3000	600	—
19–50 y (pregnant/lactating)	1000	2500	600	—

Abbreviations: m, month; y, years.

#### 6.3.2 Calcium and body mass

There is more and more evidence that a low calcium intake is linked to having more abdominal fat. Research studies have shown that calcium helps people lose weight (Pilvi et al., 2009). Importantly, the results from studies on overweight mice seem to apply to people as well. Numerous past studies (mostly cross‐sectional and retrospective cohort studies) have shown that calcium intake and muscle mass are related negatively. This link has also been seen in people of many different ages and genders. But it is important to keep in mind that diets with a low amount of calcium can have an increased overall energy concentration than similar diets with a high amount of calcium. Numerous types of research indicate that getting a lot of calcium did not make people lose weight. Even though one of these researches suggests that fat oxidation went up a lot. The review of randomized controlled trials was part of the meta‐analysis and also did not find any evidence that calcium was good for body mass. In females, a lower amount of calcium is paired with a higher amount of fat and energy (Song & Sergeev, 2012).

### 6.4 Type 2 diabetes mellitus (T2DM)

Hyperglycemia seems to be the fifth largest reason for expiry in the United States of America (USA) and a prominent factor of ailment. Despite our current approach to diabetes management and its comorbidities having progressed, it is still better to prevent the illness (Khan et al., 2019). Moreover, scientific research indicates that behaviors or other types of adjustable lifestyles are crucial for nine out of ten incidents of prediabetes. Being overweight has been measured as one of the foremost genetic susceptibility ecological threat factors for T2DM. Denying the reality that fat loss has been observed to prevent T2DM is hard to accomplish and uphold for an extended period. To avert the progression of diabetes in the 40 million American females who may be at the potential for diabetes, ecological and widely changeable lifestyle factors must be determined. The latest research shows that vitamin D and calcium balance are dangerous for various nonskeletal consequences such as neurotransmission and injuries, eczema, multiple sclerosis, and colon and tumor cells. Vitamin D and calcium were also studied to regulate potential diabetes risk (Aatsinki et al., 2019). According to a human and animal survey study, dysfunction has long been assumed to be a potential risk for hyperglycemia (Grundy, 2012).

#### 6.4.1 Insulin resistance

Vitamin D might improve insulin sensitivity by enhancing insulin signaling pathways and thus rising insulin sensitivity for amylase uptake, or passively over its participation in modulating exogenous calcium and providing optimal ca channels across cell walls and an adequate internal cytoplasmic calcium [Ca^2+^] reservoir (Muoio & Newgard, 2008). Vitamin D may boost insulin sensitivity in two directions: directly by altering insulin signal transduction and hence increasing insulin levels for glucose utilization, and indirectly by regulating extracellular calcium and giving suitable calcium channels throughout cell membranes as well as ample cytosolic calcium [Ca^2+^]. Variations in [Ca^2+^] insulin target organs might lead to impaired insulin secretion by decreasing glucose transporter‐4 functioning due to impaired insulin signaling pathways (Newsholme et al., 2014). Cross‐sectional studies had initiated links between low vitamin D levels and insulin deficiency. Most past studies had reported an adverse implication between vitamin D or calcium levels and hyperinsulinemia, albeit not all (Güdücü et al., 2014).

#### 6.4.2 The link between vitamin D levels and the risk of T2DM

In the Women's Health Study, a vitamin D supplementation of 511 IU/d or above was allied to a diminished risk of T2DM. That research, therefore, cannot compensate for alternative diabetes complications hazards or calcium usage. Throughout the Nursing Research Institute, a prospective randomized study of 83,906 women, this research team explored the correlation between vitamin D and T2DM risk (Syeda, 2018). We indicated a major inverse relation between vitamin D consumption (food + supplements) as well as the hazard of prediabetes even adjusted for age, BMI, guidelines as well as recommendations variables. Whenever food determinants, primarily magnesium and calcium, when taken into account, the relationship was diminished (Lucato et al., 2017).

#### 6.4.3 The link between calcium levels and the menace of T2DM

Poor calcium availability is frequently related to the incidence of prediabetes and metabolic disorders in observational studies (Becerra‐Tomás et al., 2014). Despite thorough multifactorial analysis, including vitamin D use, total calcium intake was found to be negatively proportionate to the occurrence of T2DM in the Nurses' Health Survey. The Black Women's Health Research, coupled with a change of roughly 59,000 women aged 21–69 years at the start, noticed a similar negative relation (Coogan et al., 2019). There' was no modification for vitamin D levels in the former research, although the effect was diminished once magnesium consumption was accounted for. After merging the information from the two groups, the aggregate (95% confidence range) for the incidence of prediabetes was 0.82 (0.72–0.93) for the maximum vs. least calcium consumption (661–1200 vs. 219–600 mg/d, correspondingly). Those analyses indicate that calcium supplementation has a serious impact (Kayaniyil et al., 2010).

### 6.5 Vitamin D, calcium, and premenstrual syndrome

Premenstrual syndrome (PMS) is the cyclical emergence of one or more signs before menstruation, which can influence the quality of life. Acid reflux, breast discomfort, anxiety, weeping episodes, sadness, weariness, decreased energy, aggression and aggressiveness, decreased appetite, and edema are common side effects. PMS is 70%–90% prevalent throughout the reproductive years. PMS is 70%–90% prevalent throughout the reproductive years. Identification involves at least one sign of physical or emotional problems recurring over three consecutive months, interfering with a job or customary hobbies, and repatriating after each menstruation (Zareef et al., 2018). According to 2015 research, women experiencing PMS had greater job interruptions. PMS women experienced greater school or work disruptions than those without PMS. Intracellularly calcium content might stimulate neurological connections, and hypocalcemia has been linked to restlessness, anxiety, and instability. Based on what we saw, we decided to see if taking 500 mg of calcium carbonate two times a day for 3 months would help reduce period pain (Kia et al., 2015).

#### 6.5.1 Position of vitamin D and calcium in PMS

Vitamin D is a hormone allied to calcium, phosphorus, and other metabolic activities. Humans get 80% of their dose of vitamin D from 7‐dehydrocholesterol on UVB radiation and 20% from food. Vitamin D has seemed to reduce PMS risks via modulating calcium, neurotransmitters, and sexual stimulants. Vitamin D promotes cell development and growth. During perimenopause, serum 25‐hydroxyvitamin D3 ([25(OH)D3] or [25(OH)D]) has been shown to change in some studies. Sexual hormones made by the ovary cause enzymes that break down [25(OH)D3]. Estradiol makes 1‐hydroxylase and 24‐hydroxylase in the liver work more quickly, which lowers the amount of 25(OH)D3 in the blood. During the luteal phase, the level of estrogen in the ovaries is highest, which breaks down 25(OH)D3 and lowers its level in the blood. So, the ovarian endogenous testosterone cyclic disestablishment of serum [25(OH)D3] metabolism could make the signs of PMS severe (Abdi et al., 2019). According to research, blood calcium levels rise during the follicular phase and fall during perimenopause. In some women's menstruation, serum calcium levels drop. Several types of research show that lack of calcium during the luteal menstrual phase can make PMS symptoms worse through emotional dysregulation, hallucinations, and irritability. So far as we understand, no literature review has looked at how vitamin D and calcium mark the improved performance of PMS symptoms. The whole analysis studied the part of vitamin D and calcium in PMS. The outcomes of some studies could help make decisions about how complementary therapies should be given to women which lower risk treatments. Also, women who eat a lot of vitamin D and calcium have a lesser chance of getting PMS than other women (Arab et al., 2019).

#### 6.5.2 The link between vitamin D receptor Fok1 polymorphism or calcium and PMS

VDR Fok1 gene polymorphism has not been studied in menopausal syndrome. PMS is a mix of symptoms and complications that arise before ovulation and ends with ovulation. During perimenopause, 80% of menstruation women worldwide have PMS symptoms. PMS is identified if at least one of the physical signs occurs in the 5 days before menstruation and at least 3 successive cycles. These clinical signs can be mental (like distress, frustrated temper tantrums, lack of energy, nervousness, uncertainty, and personality changes) or physiological (like breast tenderness, gastrointestinal discomfort, headaches, and inflammation of the extremities). Symptoms often subside within 4 days of menstruation and do not return until day 13 of the cycle. Symptoms occur without pharmaceutical treatment, hormone consumption, or drug usage. According to researchers, PMS decreases mental health and general welfare, especially among young women. PMS is unrecognized. Signs can be induced by hormonal, neurotransmitters, psychological pressures, and necessary nutrients. Fact therapies include reducing coffee intake, quitting smoking, and psychotherapy to boost self‐esteem. Anti‐inflammatory drugs, which lessen muscle cramps and chest tenderness, and antidepressants, such as serotonin reuptake inhibitors, are some of the chemotherapy drugs that have been suggested. Because hypocalcemia and PMS symptoms correlate, calcium supplements might be used as a therapy. Vitamin D levels oscillate in women at reproductive age but its link to PMS and premenstrual dysphoric disorder (PMDD) remains unknown. Ovarian hormones affect calcium and vitamin D metabolism. Estrogen normalizes the absorption of calcium, parathyroid gene expression, and hormone production, which may describe female reproductive cycle variations. Researchers discovered that FF homozygous had a mean calcium absorption that was 41.5% higher than FF homozygous and 17% higher than FF heterozygous. This means that FF homozygous may interfere with calcium absorption. So far as we know, this study is the first report of an investigation into a possible link between VDR polymorphism and PMS. This connection was chosen because of the links between vitamin D and menstrual problems, as well as the fact that there may be a hereditary linkage (da Silva Lopes & Abe, 2021).

### 6.6 Polycystic ovary syndrome and vitamin D

Vitamin D values have been accompanied by a diversity of PCOS indicators, notably glucose intolerance, subfertility, and hirsutism, in a series of investigations (Yildizhan et al., 2009). Vitamin D is assumed to take part in the establishment of PCOS by influencing gene transcription, whereas hormonal manipulation affects insulin homeostasis and ovulation (Mahmoudi, 2009; Lips, 2010). Vitamin D levels appear to be similar in women having and without PCOS (Li et al., 2011); nevertheless, reduced concentrations (Lerchbaum et al., 2012) and greater levels (Mahmoudi et al., 2010) have indeed been disclosed in women with PCOS. Plentiful investigations have found inadequate vitamin D levels in women with PCOS, with normal 25‐hydroxy vitamin D (25OHD) values fluctuating from 10 to 30 ng/mL (Muscogiuri et al., 2012), with the most (65%–80%) reporting values underneath 20 ng/mL (Holick, 2007; Li et al., 2011).

#### 6.6.1 Vitamin D levels in PCOS patients fluctuate depending on their BMI

Several investigations have found an inverse relationship among body weight (abdominal fat, BMI, and waist measures) and serum 25(OH)D concentrations in women with PCOS (Li et al., 2011), with levels reported to be 25%–55% lesser in obese women with PCOS than nonobese women with PCOS (Muscogiuri et al., 2012). Lower 25(OH)D concentrations were also observed to be strongly influenced by the amount of adiposity in women with PCOS and were not even significantly impacted by the establishment of insulin resistance, according to a current analysis (Muscogiuri et al., 2012). Elevated frequency of vitamin D insufficiency in PCOS women is probably linked to obesity because vitamin D is fat soluble, and a greater percentage of it is stored in adipose tissue in obesity, limiting bioavailability (Lagunova et al., 2009; Li et al., 2011).

**FIGURE 4:**
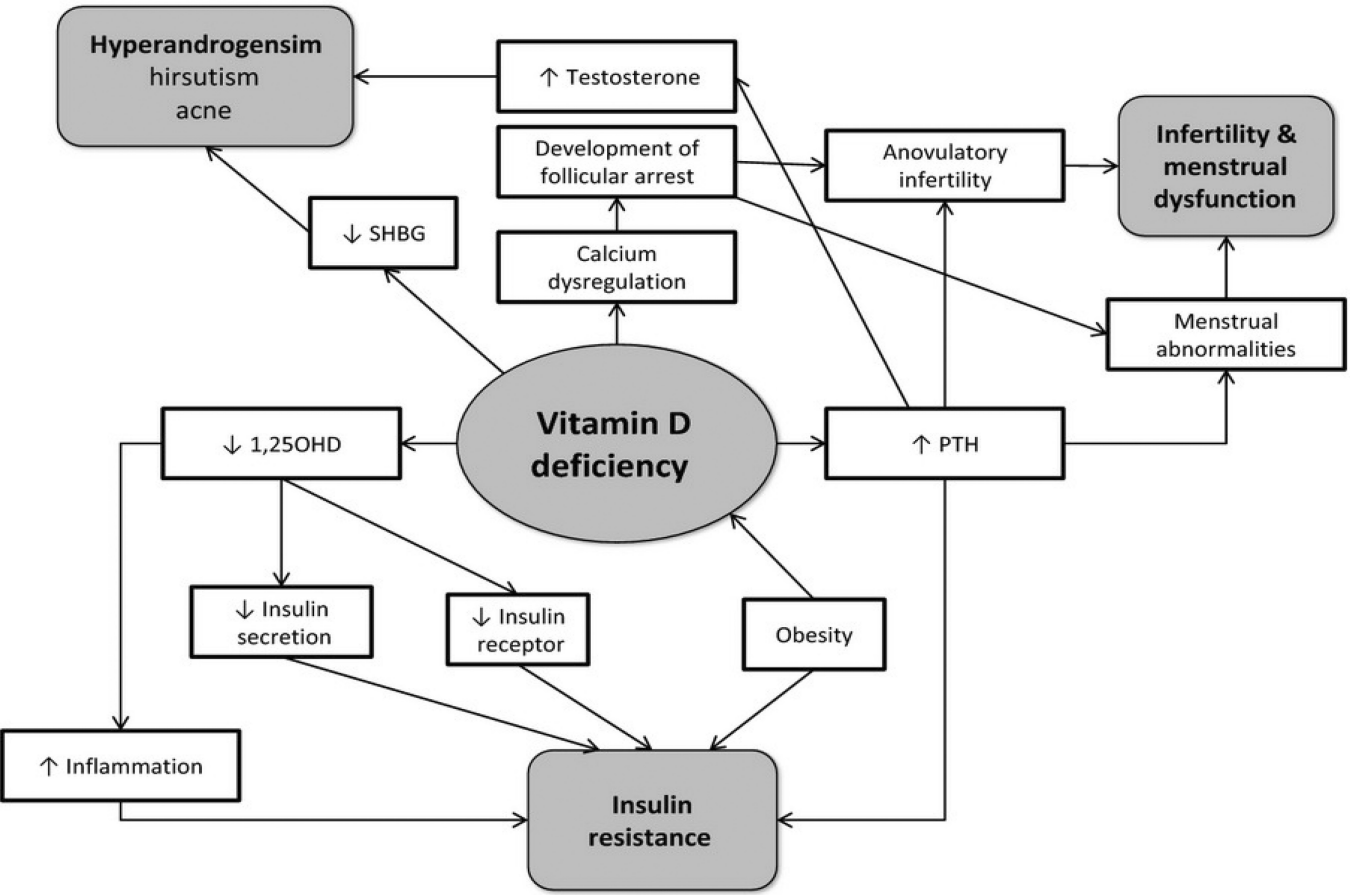
Vitamin D deficiency's involvement in the pathophysiology of PCOS (Source; Thomson et al., 2012).

Obese people, on the other side, might spend fewer hours outside in the sunlight, which can result in inadequate vitamin D construction in the skin. It is also likely that obese and nonobese people have different dietary choices and vitamin D metabolism. There was, meanwhile, one experiment that found no variation in 25(OH)D concentrations along a variety of BMIs (Zadka et al., 2018). There is currently insufficient information to conclude the cause of the discrepancy in vitamin D levels across slim and obese PCOS females (Figure 4).

#### 6.6.2 Vitamin D deficiency, PCOS etiology, and insulin resistance

There are indications that vitamin D inadequacy might perform a part in the development of insulin resistance and metabolic syndrome in PCOS (Ngo et al., 2011). Vitamin D has impacts that are regulated by both gene and cell processes. Vitamin D controls gene transcription via nuclear VDR, which is found in a variety of organs such as the bones, parathyroid glands, and the ovarian (Morgante et al., 2022). The impacts of VDRs (TaqI, BsmI, FokI, ApaI, and Cdx2 polymorphisms) on LH and SHBG concentrations (Ranjzad et al., 2011), testosterone concentrations (Wehr et al., 2011), insulin resistance, and plasma insulin concentrations have all been associated with the pathophysiology of PCOS (Wehr et al., 2011).

Elevated PTH is connected with PCOS, anovulation infertility, and elevated testosterone. Vitamin D insufficiency rises PTH generation that is mediated by serum calcium and vitamin D concentrations. It has been recommended that a fusion of vitamin D insufficiency and dietary calcium deficit might well be primarily accountable for the menstrual anomalies affiliated with PCOS (Akbari et al., 2018); moreover, it has also been proposed that vitamin D adequacy is more essential than elevated calcium ingestion in sustaining preferred PTH levels (Steingrimsdottir et al., 2005). Furthermore, current research indicated that a lesser calcium consumption was linked with greater serum testosterone levels in women with PCOS (Lerchbaum et al., 2012), indicating that low calcium consumption could influence the hormonal imbalance seen in PCOS (Kinuta et al., 2000).

VDRs are involved in the synthesis of estrogen in the ovary. Vitamin D influences estrogen production by preserving extracellular calcium homeostasis and directly regulating the transcription of the aromatase gene (Kinuta et al., 2000). VDR‐null animals showed reduced aromatase activities in ovaries and poor folliculogenesis (Kinuta et al., 2000), while vitamin‐D‐deficient mice had lower fertility levels (Halloran & Deluca, 1980). The increase of estrogen and progesterone synthesis by 1,25‐dihydroxy vitamin D3 in the human ovarian cortex and the absence of impact on testosterone generation possibly is understood by vitamin D enhancement of aromatase activity (Parikh et al., 2010). PCOS follicles exhibited lower aromatase expression of genes than counterparts and exhibited higher LH concentrations yet lower follicle synthesis of progesterone and estrogen by early follicles, probably due to the hyper luteinized environment of PCOS follicular. Vitamin D insufficiency may increase PCOS signs as a consequence of such impacts (Sander et al., 2011).

Whereas the specific mechanism underpinning the link between vitamin D and insulin resistance remains unknown, several cellular and molecular theories have been postulated to elucidate the association. 1,25‐dihydroxy vitamin D (1,25OHD), a physiologically functional version of vitamin D, may improve insulin activity by boosting insulin manufacturing and secretion, elevating insulin receptor transcription, or suppressing pro‐inflammatory cytokines that are thought to cause insulin resistance (Teegarden & Donkin, 2009). Vitamin D possibly will potentially improve insulin sensitivity by boosting the localized synthesis of 25OHD that contributes to transcriptional control of genes or reducing blood concentrations of PTH (Teegarden & Donkin, 2009).

In contrast, a recent investigation that employed the gold benchmark for determining peripheral insulin sensitivity discovered that vitamin D insufficiency was linked to the existence of obesity rather than insulin resistance (Muscogiuri et al., 2012). While there is a strong indication linking vitamin D levels to insulin sensitivity (Teegarden & Donkin, 2009), more study is required to entirely explicate the processes.

#### 6.6.3 Calcium and PCOS

Numerous studies have shown how calcium affects ovulation, follicular development, and the pathophysiology of PCOS (Anagnostis et al., 2013; Brzozowska & Karowicz‐Bilińska, 2013). It is thought that their function in this area is unaffected by insulin resistance. Therefore, it appears that calcium plays a significant impact in the transition of testosterone to estrogens in granulosa cells, which ultimately created a balanced amount of androgen and estrogen in PCOS sufferers (Das et al., 2008).

Numerous research has shown that supplements may have positive impacts on PCOS indicators like fertility problems, normal menstrual periods, BMI, glucose intolerance, and hyperandrogenic traits (Dehghani Firouzabadi et al., 2012; Galusha, 2013). Galusha recently concluded in a systematic review that, despite the premise that several research has documented its beneficial benefits in this area, there are several debates surrounding it and the reliability of the data is not sufficient (Fritz & Speroff, 2011).

### 6.7 Multiple sclerosis

Multiple sclerosis (MS) is a central nervous system (CNS) inflammatory neurodegenerative illness that causes severe neurological impairment. The pathophysiology of MS remains still completely unclear, that is why treatment for MS is ineffective (Hauser & Oksenberg, 2006). Vitamin D inadequacy is often observed as a possible cause of MS. Myelin sheath destruction, oligodendrocyte, axonal, and neuronal damage are all symptoms of multiple sclerosis. MS affects more than two million people all over the globe. Paresthesia, ataxia, cognitive impairment, as well as eyesight, and mobility loss, are all signs of the condition. After numerous periods of remission and recurrence cycles, most MS patients have a bad prognosis. A lot of ecological factors, particularly vitamin D insufficiency, the background of Epstein–Barr virus (EBV) infection, and tobacco are also believed to have a part in MS progression (Ascherio et al., 2010).

#### 6.7.1 The level of vitamin D and how it affects the threat of MS

The premise that individuals living further from the tropics absorb lower UVB light and thus have greater vitamin D insufficiency and a greater menace of MS is a considerable clinical link between MS and vitamin D. The discrepancy between the benefits of ultraviolet irradiation and possessions of vitamin D on MS jeopardy is just adequately controlled for an observational study since ultraviolet exposure can also limit the formation of MOG EAE. The concept that those who live farther away from the equator absorbed less ultraviolet radiation has more vitamin D deficit, and so has a higher risk of MS, is a significant clinical relation between MS and vitamin D (Sintzel et al., 2018). Because ultraviolet light can also reduce the production of MOG EAE, the mismatch between the advantages of ultraviolet light and the impacts of vitamin D on MS danger is only barely sufficiently corrected for in epidemiological studies. Several findings reported that persons with fewer 25(OH)D levels had a higher chance of getting MS, suggesting that 25(OH)D levels do augment MS chance. In a Scandinavian analysis, kids of women with 25(OH)D levels of less than 12.02 ng/mL (30.05 nmol/L) throughout prenatal had a nearly two‐fold probability of gaining MS.

In conclusion, newborns in the bottom quintile (8.30 ng/mL) seemed to have the greatest risk of having MS; however, newborns in the top quintile (18.9 ng/mL or 49 nmol/L) had the least. This study found that participants not only had reduced 25(OH)D levels than normal participants in the 24‐month time leading up to the start of clinically separated condition but that 25(OH)D levels also slightly decreases as another clinical feature of MS approaches.

#### 6.7.2 Vitamin D supplements and treatment for MS relapses

Researchers assessed whether 1,25(OH) 2D3 enhances the effectiveness of methylprednisolone pulse stimulation for the prevention of MS exacerbations due to the immunological system by which 1,25(OH) 2D3 enhances the effectiveness of corticosteroids. Researchers determined that 1,25(OH) 2D3 augmented the appearance of methylprednisolone‐induced mortality in human and mouse CD3^+^ T cells via enhancing glucocorticoid receptor protein levels. In vivo, the treated group suppressed actual MOG35–55 EAE disease incidence substantially. Additionally, persons with steroid‐resistant MS had relatively lower 25(OH)D contents in two separate cohorts. Since no health benefits were detected in mTORc1‐deficient rodents, yet therapy of wild‐type individuals with mTOR inhibitors leads to simultaneous glucocorticoid actions, the most logical cellular route to explain this observation is mTOR inhibition (Munger & Ascherio, 2011).

#### 6.7.3 The function of calcium in MS

Throughout a prolonged period, the goal of MS investigation and therapy was to lessen immune cell infiltration into the CNS, which prompted the creation of numerous dimethyltryptamine (DMT). However, the development of neuroprotective approaches is urgently required to stop the long‐term progression of illness, impairment, and the associated socioeconomic burden. In MS and other neurological illnesses, numerous therapy methods following similar lines have previously been explored and are presently being evaluated. Although several experimental types of research employing cell culture and mouse models yielded hopeful findings, solely the medicine siponimod has reached the point of clinical use for the management of secondary progressive multiple sclerosis (SPMS) patients due to its subsequent authorization in the US and Europe (Trapp & Nave, 2008).

The reasons why calcium could be a viable therapeutic strategy for MS neuroprotection are reviewed and discussed below.

##### Calcium and Excitotoxicity

Excitotoxicity is known to happen in pathological circumstances, and it has been linked to both MS and experimental autoimmune encephalomyelitis (EAE) (Rajda et al., 2017). The pathogenic route of excitotoxicity involves α‐amino‐3‐hydroxy‐5‐methyl‐4‐isoxazole propionic acid (AMPA) and kainic acid receptors (KARs) and blocking of these receptors can lessen the intensity of EAE. One explanation for the elevated glutamate receptor functioning observed in EAE is that T cells either directly interact with the receptors or boost glutamate transmission by releasing tumor necrosis factor (TNF‐α) (Gentile et al., 2020). Additionally, TNF‐α promotes the release of glutamate from microglia, which may exacerbate cause excitotoxicity (Thomas et al., 2014). Another theory of excitotoxicity proposes that glial cells' glutamate absorption is changed. Interleukin 17, a cytokine associated with EAE and MS, has been found to inhibit glutamate absorption in astrocytes while increasing calcium‐dependent glutamate release (Kostic et al., 2017). Another research found that AMPA antagonists might inhibit the disruption of glutamate absorption in oligodendrocytes, which could be caused by chemicals (Domercq et al., 2005). In locations where glutamate absorption was disrupted, activated microglia accumulated in the postmortem brains of MS patients (Vercellino et al., 2007). Increased glutamate levels can, in return, produce an elevation in intracellular calcium concentrations, which, once more, leads to excitotoxicity through reactive oxygen species (ROS) generation and calcium overflow in mitochondria (Joshi et al., 2015). Regulating the transient receptor potential melastatin 2 (TRPM2) channels, which mediates microglia stimulation and is highly susceptible to oxidative stress, may lessen excitotoxic effects (Malko et al., 2019).

##### The intactness of the BBB and calcium

Anatomically, the BBB is made up of astrocytes, endothelial cells, and smooth muscle cells, producing a protective natural border for the brain. Additionally, it affects calcium homeostasis. The actin cytoskeleton within the cells as well as the strong and adhesive connections that existent amongst the endothelial cells of the BBB are reliant on the intracellular and extracellular calcium levels (Abdullahi et al., 2018; González‐Mariscal et al., 2018). Following injury, endothelial cells' G‐protein‐coupled receptors (GPCR) and inositol trisphosphate (IP3) signaling, following store‐operated calcium entry (SOCE) activation, and a direct calcium influx through calcium channels all contribute to an elevation in intracellular calcium levels. As a result, the actin cytoskeleton and tight junctions are reorganized and/or modified, which causes BBB leakage (Stamatovic et al., 2016). It is interesting to note that cannabinoid CB2 receptor antagonists have been demonstrated to improve the establishment of adherent junctions and decrease BBB leakiness (Ramirez et al., 2012). Additionally, in an animal model of strokes, stimulation of the calcium/calmodulin‐dependent protein kinase (CaMK) has been shown to improve BBB restoration, which may reduce immune cell infiltration (Sun et al., 2019).

### 6.8 Breast cancer

Amongst females' breast cancers are the most recurrent kind of cancer in 161 regions and the prime cause of cancer fatalities in 98 countries. Gender, family background, breast tissue thickness, menstruation, obesity, alcohol, and known heritable factors such as BRCA abnormalities are all known breast cancer risk factors. Vitamin D receptor (VDR) genes have recently been exposed and implicated in the development of breast cancer (Slattery, 2007). Numerous genetic breast cancer variants are recognized such as luminal A and B (which account for 50% of breast cancer incidences) basal‐like or triple‐negative (10%–20% of tumor cases) and HER2‐enriched (which stands for 10%–20% of tumor cases) 10%–15% of cases (Lam et al., 2014).

According to modern research, higher intakes of calcium and vitamin D may influence lowering the threat of colon and breast cancers even in the absence of a high‐fat diet (Chen et al., 2010). Increased dietary calcium and vitamin D in a high‐fat diet led to unfavorable effects on the mammary gland and numerous other tissues. According to studies from our research, increased dietary calcium and Vitamin D may also have a part in cancer anticipation of those malignancies (Feldman et al., 2014). In the condition of a high fatty acid intake, further investigation determined that vitamin D and phosphate interact with calcium intake. In the occurrence of low dietary calcium and phosphate increasing doses of dietary vitamin D suppressed tumor, but it was less efficient with a rising calcium intake (Carmeliet et al., 2015). Particularly as per this concept, high dietary fat during rapid growth leads to a rise in the influx of circulating lipids into the breast tissue. These lipids might elevate their availability in mammary cells, especially minor duct epithelial cells, where abnormalities are more possible to occur. Sometimes the oxidation of systemic lipids generates free fatty acids for transport across nearby fat cellular sheaths (Le et al., 2009).

Interfaces among parathyroid hormone, calcitonin, 1,25 di‐OHD3‐controlled food absorbing excretion related to combining with consumed food materials and wastes in urine, and sweating are all vital physiologically to uphold calcium blood levels contained by a limited array. On the other end, rising 1,25 di‐OHD3 concentrations in the blood promotes cellular calcium absorption from the bloodstream. Inadequate vitamin D may, thus, increase the possibility of circulating lipids to stimulate breast cancer growth and reduce the individual physical ability to raise 1,25 di‐OHD3 (Shamsi et al., 2020).

Amounts of vitamin D were slightly reduced in breast cancer patients related to good controls. Moreover, low Vit D concentrations were prevalent at the time of breast cancer classification and were related to a poor diagnosis in 93% of women with a vitamin D concentration less than 20 ng/mL develop metastases and 74% die from chronic cancer. When comparing individuals with early‐stage breast cancer to those with localized progressive or metastatic tumors, the 25(OH)D levels are advanced in those with early‐stage breast cancer (de La Puente‐Yagüe et al., 2018).

Vitamin D modulates the activation of over 60 genes within cells which have antiproliferative, PR differentiating, antimetastatic, and inducing apoptosis characteristics. Reduction in blood vitamin D levels enhances cellular proliferation, neo‐angiogenesis, and tumor progression, and VDR mutant rodents acquired higher preneoplastic glandular lesions. According to the latest study, vitamin D promotes autophagy including both healthy mammary gland and basal tumor cells which could simplify the association between both vitamin D levels and breast cancer (Costa et al., 2020).

Moreover, recent research has guided that vitamin D and calcium might take part in the advancement of malignancy. Thus, functionally active vitamin D like 1,25 di‐OHD3 and 1‐alpha‐hydroxycholecalciferol (1 alpha‐OHD3) have demonstrated strong evolutionary divergence capabilities on tumor cell lines from various tissues (Thorne & Campbell, 2008). The control of epidermal growth factor transmitters and morphologic variations in cells could be engaged in the variability actions of 1,25 di‐OHD3 on tumor cell lines. Chemotherapy with the synthesized analog 1‐a‐OHD3 substantially suppressed tumor formation in mice caused by methyl nitrosourea. Subsequent research on individual breast tumors and carcinoma cells indicated that 1,25 di‐OHD3 receptors are identified in over 80% of breast cancer individuals and that1,25 di‐OHD3 limits growth and stimulate development in a multicellular organism in vitro (Mehta et al., 2000). Persons with cancers that express the 1,25 di‐OHD3 receptors had a substantially longer illness life than one with malignancies that did not acknowledge the receptor. In general concentrations of 1,25 di‐OH D3 might, therefore, inhibit receptor‐positive tumors (Newmark & Lipkin, 1997).

## 7 ROLE OF VITAMIN D AND CALCIUM ON POSTMENOPAUSE STAGE

### 7.1 Osteoporosis

Osteoporosis is a chronic, progressive bone ailment that reduces bone mineral density and disrupts bone microarchitecture. Females with osteoporosis are more likely to fracture from stressors that would not affect a nonosteoporotic person. Osteoporosis is especially common in postmenopausal women since the reduction in ovarian estrogen promotes bone loss and bone remodeling. Vitamin D and calcium supplements have been studied for their influence on osteoporosis and fracture hazards in postmenopausal women (Johansson et al., 2021; Rathnayake et al., 2019).

1,25(OH) 2D boosts calcium absorption from stomach. Vitamin D insufficiency causes secondary hyperparathyroidism, bone loss, osteoporosis, fractures, mineralization abnormalities, osteomalacia, and muscular weakness, which causes falls and fractures. Vitamin D may reduce bone turnover and promote bone density. Very high vitamin D dosages once a year may be harmful. Bisphosphonate‐treated osteoporosis patients should get a vitamin D and calcium supplement unless vitamin D is replete (Polzonetti et al., 2020; Kebaetse et al., 2021).

Calcium supplementation is a key aspect of preventing and treating postmenopausal osteoporosis, but recently its influence on bone mass becomes obvious. Calcium supplements reduce postmenopausal bone loss by 30%–50% (Salmanpour et al., 2016). Calcium supplementation's effect on postmenopausal fractures remains unknown. Frail elderly often lack vitamin D, especially in places where diet is not fortified. Vitamin D treatment reduces hip fractures. It remains unclear how calcitriol and alfacalcidol affect postmenopausal osteoporosis (Bhadada et al., 2021). Published evidence on the possessions of these chemicals on bone density or breakage rate is conflicting (Hao et al., 2020; Quattrini et al., 2021).

### 7.2 Postmenopause phase

To answer this topic, vitamin D and calcium thresholds are examined in which bone health is damaged. This should be based on the link between nutritional consumption and skeletal health. Low levels of 25‐hydroxyvitamin D (25[OH]D) are associated with increased release of parathyroid hormone (PTH), which promotes bone loss elderly through augmented bone resorption (Charoenngam et al., 2019). Between 30 and 100 nmol/L of circulating 25(OH)D is needed to uphold regular PTH levels. Depending on the criterion utilized, approximations of vitamin D deficit in communities vary substantially (Mendes et al., 2020). In a study of 8500 postmenopausal, osteoporotic European women, 80% had vitamin D deficiency when the threshold was 80 nmol/L and 32% when it was 50 nmol/L reported by Polzonetti et al. (2020).

Recent studies found inadequate evidence to warrant supplementing the diets of nonosteoporotic women within the general population. Eastell et al. (2019) stated that shared calcium and vitamin D treatment in postmenopausal females has indicated a decrease in fracture risk, assuming acceptable patient conformity. The group agreed that females in jeopardy of osteoporosis and those with the disease should take calcium and vitamin D supplements. Vitamin D dosage should ensure circulatory 25(OH)D values approach 50 nmol/L (Zittermann, 2017). Younger women with nutritional insufficiency and fracture risk should also be supplemented. From a health economic viewpoint, supplementing with vitamin D and calcium could be defensible in females under 65 with calcium scarcity since the combination could minimize bone turnover. Those at higher risk may benefit from bigger dosages (Wimalawansa, 2018).

Vitamin D supplementation must guarantee serum 25(OH)D levels reach the threshold level to be beneficial. 400 IU per day of vitamin D does not diminish fracture risk, according to studies (Liu et al., 2020). Oral doses of 700–800 IU per day or 100,000 IU every 3 months had a positive effect on preventing fractures, but an intramuscular dose of 300,000 IU per year had a mixed effect. This suggests that supplementation works best for people with osteoporosis if it is taken by mouth either every day or every 3 months and if it is taken every day, it should be more than 700–800 IU (Polzonetti et al., 2020).

## 8 CONCLUSION

Calcium is very crucial for adequate health in females. For proper metabolism, calcium needs vitamin D which plays its key role in synthesizing protein carriers required in gut absorption and renal reabsorption of calcium. Recent studies have proved that hypocalcemia results in increased lipogenesis that results in obesity which is the main culprit in a variety of metabolic disorders in females. Females are more at risk for calcium deficiency due to the high requirement for calcium in a specific event of their life like pregnancy, lactation, and the postmenopausal phase. This review has related different female disorders with calcium and vitamin D deficiency intending to provide enough knowledge in the prevention and treatment of these maladies.

There is a lot of uncertainty about the relationship between vitamin D, calcium, metabolic syndrome, its components, CVD, and death. Although researchers have not been consistent in their conclusions, we have the perception that hypovitaminosis D and inadequate calcium consumption are likely connected with all of the aforementioned outcomes. But it remains unclear what this link looks like when it comes to subgroups (such as gender, age groups, ethnicity, etc.). However, there seems to be enough information to make it imperative to understand how inadequate calcium consumption, hypovitaminosis D, and metabolic syndrome are related. This implies that even a slight reduction in CVD risk following calcium and vitamin D treatment will result in significant overall benefits. The relative safety and low cost of supplements may make vitamin D and calcium replenishment more cost‐effective.

The need is for well‐planned prospective and interventional trials. They must have the power to investigate subgroups, risks, and benefits at various serum vitamin D and calcium concentrations, as well as replacement regimens, in addition to assessing the overall benefit. Clear recommendations about vitamin D and calcium supplementation in individuals with metabolic syndrome components can only be made after these data are made available.

## AUTHOR CONTRIBUTIONS

**Aftab Ahmed:** Writing – original draft (equal). **Muhammad Awais Saleem:** Writing – original draft (equal). **Farhan Saeed:** Supervision (equal). **Muhammad Afzaal:** Conceptualization (equal); data curation (equal). **Ali Imran:** Investigation (equal); resources (equal). **Sidra Akram:** Resources (equal); software (equal). **Muzzamal Hussain:** Writing – review and editing (equal). **Aqsa Khan:** Validation (equal); visualization (equal). **Entessar AL Jbawi:** Supervision (equal).

## FUNDING INFORMATION

No funding was included in this work.

## CONFLICT OF INTEREST STATEMENT

The authors declare that they have no conflict of interest.

## DECLARATION

The work described has not been published before. It is not under consideration for publication elsewhere.

## Data Availability

The data that support the findings of this study are available on request from the corresponding author.
